# The effects of graft shrinkage and extrusion on early clinical outcomes after meniscal allograft transplantation

**DOI:** 10.1186/s13018-018-0892-0

**Published:** 2018-07-20

**Authors:** Jae-Hwa Kim, Soohyun Lee, Doo Hoe Ha, Sang Min Lee, Kyunghun Jung, Wonchul Choi

**Affiliations:** 1 Department of Orthopaedic Surgery, CHA Bundang Medical Center, CHA University, 351 Yatap-dong, Bundang-gu, Seongnam-si, Gyeonggi-do Republic of Korea; 2 Department of Radiology, CHA Bundang Medical Center, CHA University, 351 Yatap-dong, Bundang-gu, Seongnam-si, Gyeonggi-do Republic of Korea

**Keywords:** Meniscus allograft transplantation, Extrusion, Shrinkage

## Abstract

**Background:**

Graft shrinkage or radial extrusion is a reported complication after meniscus allograft transplantation (MAT). Whether shrinkage or extrusion progress after surgery and whether they are associated with the clinical outcome of MAT remain debatable. In this study, graft shrinkage and extrusion were measured in the coronal and sagittal planes using serial postoperative magnetic resonance imaging (MRI). The purpose of this study was to evaluate if graft shrinkage or extrusion is correlated to the clinical outcome of MAT.

**Methods:**

MRIs acquired at 3 and 12 months postoperatively in 30 patients (21 men and 9 women) who underwent MAT (6 medial and 24 lateral menisci) from 2010 to 2016 were analyzed. Two orthopedic surgeons and two musculoskeletal specialized radiologists each performed the MRI measurements. Allograft shrinkage was measured by the width and thickness of the graft at the coronal and sagittal planes. To determine the graft extrusion, distances between the proximal tibia cartilage margin and the extruded graft margin were measured in both coronal (either lateral or medial) and sagittal (both anterior and posterior) plane and relative percentage of extrusion (RPE) were calculated. Subjective International Knee Documentation Committee (IKDC) scores at 12 months were evaluated as a clinical outcome measurement, and correlations between shrinkage or extrusion of allograft and IKDC score were analyzed.

**Results:**

In the coronal plane, radial RPE averaged 43.6% at postoperative 3 months, but there was no significant progression of extrusion at 12 months (average 42.0%) (*P* = 0.728). In the sagittal plane, there were no significant progressions of anterior and posterior RPE (*P* = 0.487 and 0.166, respectively) between postoperative 3 and 12 months. Shrinkage was calculated by multiplying the width and height of the three sections and summing these values. There was no significant progression of shrinkage between postoperative 3 and 12 months (*P* = 0.150). RPE in the radial (*R* = 0.147, *P* = 0.525), anterior (*R* = 0.249, *P* = 0.264), and posterior (*R* = 0.230, *P* = 0.315) directions and shrinkage (*R* = 0.176, *P* = 0.435) were not correlated to IKDC score at postoperative 12 months.

**Conclusions:**

In the coronal and sagittal planes, extrusion and shrinkage did not progress from 3 months to 1 year. Extrusion and shrinkage had no correlation with early clinical outcomes. This finding suggests that graft extrusion or shrinkage may be not a great concern especially in early postoperative period of MAT, and multiple, serial MRI may be not necessary.

## Background

The meniscectomized knee is associated with early onset of knee osteoarthritis due to a decrease in tibiofemoral contact area and an increase in joint contact pressures, especially among people who are physically active [1–4]. When treating meniscus injury patients, efforts are made to preserve the meniscus by meniscal repair or leave the meniscus as much as possible to prevent degenerative arthritis. However, not all meniscal tears can be repaired or saved, and total meniscectomy is often unavoidable. To address the problems of meniscectomized patients, there are ongoing efforts to develop techniques for meniscus regeneration or meniscus scaffold using tissue engineering strategies [5–7]. However, most of them are preclinical studies and evidences are limited to be a standard treatment option. On the other hand, meniscal allograft transplantation (MAT) has become an alternative treatment option in relatively young and active, but symptomatic, meniscectomized patients. Although evidence of cartilage protection after MAT and long-term studies are still insufficient, studies on MAT have shown pain reduction and functional improvement [8–10].

One of the known problems of MAT is extrusion or shrinkage of allograft. Several studies have reported that extrusion and shrinkage occur at various degrees after MAT by arthroscopic findings or analyses of serial magnetic resonance imaging (MRI) data after surgery [9, 11, 12]. In addition, extrusion and shrinkage of the allograft after MAT may be a biomechanical disadvantage of the knee joint due to ineffective positioning of the allograft [13]. Therefore, adequate anatomic restoration and accurate sizing of the allograft are important for the transplant to function appropriately [14–17]. Whether extrusion or shrinkage progresses with time after surgery and whether they are associated with clinical outcome of MAT remain unclear. Moreover, graft extrusion has been evaluated mainly from the coronal plane, but rarely in the sagittal plane.

The purpose of this article is to evaluate whether extrusion or shrinkage of meniscal allograft progresses during short-term follow-up and to find out if the change of graft position or volume affects the clinical outcome. It was hypothesized that extrusion and shrinkage of the graft do not progress or affect the clinical outcome during short-term follow-up period.

## Methods

Clinical and radiographic data of consecutive 50 patients who underwent MAT between 2010 and 2016 were prospectively collected. This study protocol was approved by our institutional review board, and informed consent was acquired from each patient. The indications for MAT were age 18 ≤ years ≤ 50, history of prior subtotal or total meniscectomy, persistent localized pain that did not resolve even after conservative treatment, well-aligned mechanical axis of lower extremity, correctable ligamentous stability, relatively healthy cartilage status (Outerbridge grade II or less), and preserved joint space (> 2 mm) on a 45° of flexion weight-bearing posteroanterior radiograph. Contraindications for MAT were complete disappearance of the joint space or more than 5° of mechanical axis deviation or uncorrected joint instability.

MRI scans acquired using a SIGNA™ HDxt 3T apparatus (GE Healthcare, Waukesha, WI, USA) were performed at 3 and 12 months postoperatively. The acceptable time limit for MRI at each time point was within 1 month. Patients were excluded if the time was exceeded. Among the 50 patients, 16 patients were excluded because they did not receive MRI according to the schedule. Also, one patient who underwent anterior cruciate ligament reconstruction at the same time as the MAT and three patients who underwent anterior cruciate ligament reconstruction before the MAT operation were excluded. Finally, 30 patients were included in the study.

The 30 patients consisted of 21 males and 9 females with an average age of 35 years (range, 18 to 50). The mean time interval between meniscectomy and MAT was 22 months (range, 6 to 74). Among them, 6 received medial and 24 received lateral meniscal transplantation (Table 1).

**Table 1:** Demographics of patients

	Patients (*n* = 30)
Age (years)	35 (18–54)
Gender (male:female)	21:9
Body mass index (kg/m^2^)	25.5 ± 1.7
Height (cm)	167.7 ± 5.8
Weight (kg)	71.8 ± 7.1
Lateral:medial MAT	24: 6
Time from meniscectomy (months)	15.8 ± 19.3

### Preoperative planning

Plain radiographs were used to measure meniscal dimensions before surgery using the Pollard method [18]. In order to minimize the magnification error, a 100-mm radiopaque rod was attached to the lateral epicondyle of the femur for anterior-posterior radiograph and perpendicularly to the center of the patella for lateral images. Fresh-frozen allograft of size within 10% of the measured value was prepared.

### Surgical technique

All MAT procedures were performed by a single surgeon. Before the operation, the allografts were thawed in normal saline solution at room temperature. Medial menisci were transplanted using the double bone plug technique, and lateral menisci were transplanted using the keyhole technique [19, 20]. Diagnostic arthroscopy was first performed to check the states of meniscus, ligaments, and cartilage. The remaining meniscus was removed to within 1–2 mm of the peripheral rim, and a bleeding bed was made using a shaver. For medial meniscus, tibial tunnels were reamed over the guidewires positioned in the anatomic anterior and posterior horn attachments. Each bone plug was anchored with a non-absorbable suture in advance. Three vertical mattress sutures were placed in the posterior horn, and two vertical mattress sutures were placed in the anterior horn at 5 mm intervals using inside-out repair technique. For the lateral meniscus, a keyhole slot parallel to the posterior tibial slope was made just under the lateral tibial spine. After the graft was introduced into the joint through the anterior mini-arthrotomy site, inside-out meniscal suture fixation was performed at 5 mm intervals.

### Postoperative care

Immediately after surgery, patients were encouraged to perform quadriceps set and calf pumping as much as possible and straight leg raising exercise was begun 1 day after surgery. Two days after the operation, patients started passive knee range-of-motion exercise using continuous passive motion machine with hinged knee brace on. The goals were to achieve full extension within 1 week, 90° of flexion within 3 weeks, and 120° of flexion at 6 to 8 weeks. Toe-touch weight bearing with crutch was allowed up to 3 weeks and gradually increased to 50% of body weight until 6 weeks. Patients were then allowed full weight-bearing without crutches and hinged knee braces when one leg squatting was possible. Engaging in heavy exercise and competitive sports was prohibited until postoperative 1 year.

### Evaluation criteria

**Fig. 1:**
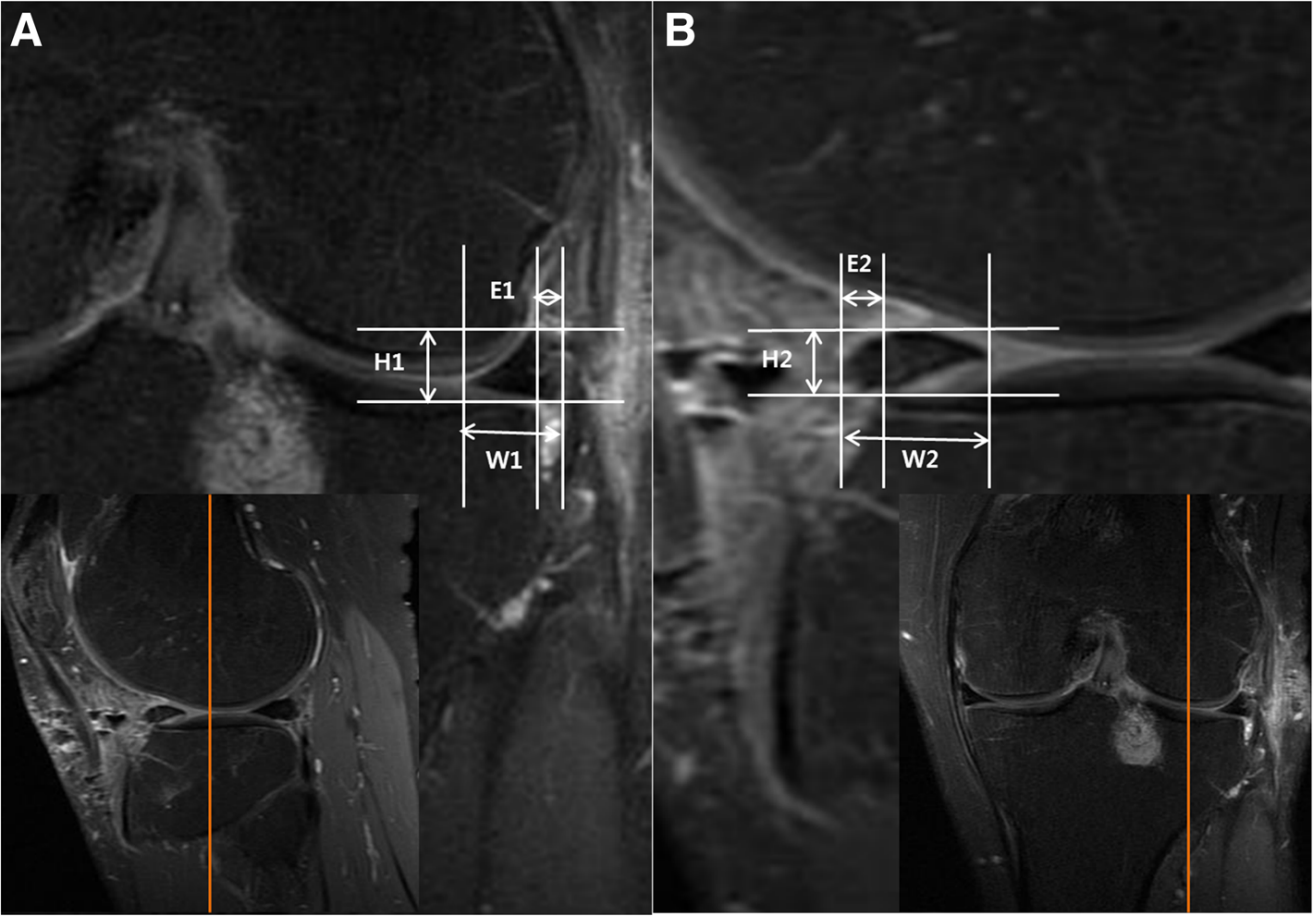
Measurements of graft extrusion or intrusion in the mid-sagittal plane and mid-coronal plane. From the mid-coronal knee MRI section (**a**), the radial extrusion (E1) was measured as the distance between the outer edge of the articular cartilage of the tibial plateau and the outer edge of the allograft. Also, the height (H1) and the width (W1) of the graft were measured. The radial relative percentage of extrusion (RPEr) was defined as the percentage of the width of extrusion relative to the width of the entire allograft (E1/W1 × 100). Similarly, the anterior relative percentage of extrusion (RPEa) was calculated after measuring the anterior extrusion (E2) and graft width (W2) from the mid-sagittal section (**b**). Graft height in sagittal section (H2) was also measured. Extrusion was expressed as a positive value and intrusion as a negative value

All the radiographic measurements were done by two orthopedic surgeons and two musculoskeletal radiology specialists. One of the observers (SL) measured twice at 1-month interval to evaluate intra-observer reliability. Picture Archiving Communication System (Marosis, Infinity, Seoul, Republic of Korea) was used for the measurements. Anterior and posterior extrusions were measured in the mid-sagittal section, and the radial extrusion was measured in the mid-coronal section. The mid-coronal and mid-sagittal sections were pre-determined under the agreement of observers and each observer measured from the same section. The extrusion was defined as the distance between the outer edge of the articular cartilage of the tibial plateau and the outer edge of the meniscus. The relative percentage of extrusion (RPE) of anterior (RPEa), posterior (RPEp), and radial (RPEr) directions were calculated to evaluate the degree of extrusion (Fig. 1). The intruded meniscus was described as negative value. Shrinkage was evaluated by measuring the height and width of the meniscus in each sections and multiplying them to estimate the volume. Subjective International Knee Documentation Committee (IKDC) scores at preoperative and postoperative 1-year periods were recorded as clinical outcome measurements.

### Data analyses

Statistical analyses were performed using SPSS 16.0 for Windows (SPSS, IBM, Chicago, Illinois, USA), with *P* < 0.05 considered statistically significant. The inter- and intra-observer reliabilities of measurements were analyzed by intra-class correlation coefficient (ICC). Change of anterior, posterior, radial RPE and shrinkage between 3 and 12 months after MAT were analyzed by paired *t* test. Also, the difference between preoperative and postoperative 1 year subjective IKDC scores was compared using paired *t* test. The differences of RPE and shrinkage between the medial and lateral menisci were analyzed by Mann-Whitney *U* test. Correlations between demographics or radiographic measurements and IKDC score were analyzed using Pearson’s correlation analysis.

## Results

Inter- and intra-observer reliabilities of extrusion and shrinkage were excellent (Table 2). There were no significant progressions of anterior (*P* = 0.487) and posterior RPE (*P* = 0.166) between postoperative 3 and 12 months. Also, in the coronal plane, there was no significant progression of radial RPE between postoperative 3 and 12 months (*P* = 0.728). Shrinkage was calculated by multiplying the width and height of the three sections and adding them together, and there was no significant progression of shrinkage between postoperative 3 and 12 months (*P* = 0.150) (Table 3).

**Table 2:** Intra-class correlation coefficients (ICC) for measurement of relative percentage of extrusion (RPE) and shrinkage

	Months	Inter-observer ICC	Intra-observer ICC
RPEa^a^	3	0.897 (0.806–0.946)	0.928 (0.897–0.952)
	12	0.871 (0.758–0.936)	0.947 (0.934–0.959)
RPEp^b^	3	0.900 (0.812–0.950)	0.917 (0.895–0.924)
	12	0.863 (0.740–0.933)	0.888 (0.796–0.902)
RPEr^c^	3	0.954 (0.914–0.977)	0.966 (0.953–0.978)
	12	0.921 (0.851–0.962)	0.918 (0.897–0.926)
Shrinkage	3	0.935 (0.878–0.968)	0.976 (0.966–0.990)
	12	0.914 (0.839–0.957)	0.944 (0.929–0.955)

Values are expressed as ICC and 95% CI in parentheses

^a^*RPEa* relative percentage of extrusion at anterior meniscus

^b^*RPEp* relative percentage of extrusion at posterior meniscus

^c^*RPEr* relative percentage of extrusion at radial meniscus

**Table 3:** Progression of relative percentage of extrusion (RPE) and shrinkage

	3 months	12 months	*P*
RPEa	40.3 ± 4.6	41.7 ± 5.4	0.487
RPEp	− 27.7 ± 6.1	− 21.2 ± 5.6	0.166
RPEr	43.6 ± 3.1	42.0 ± 3.7	0.728
Shrinkage	172.0 ± 8.2	180.6 ± 11.5	0.150

Values are expressed as mean ± SD unless otherwise indicated

Preoperative subjective IKDC score (average 37.7 ± 12.4 points, range 18 to 50 points) significantly improved after 1 year (average 69.0 ± 11.9 points, range 50 to 92 points) (*P* < 0.001).

**Fig. 2:**
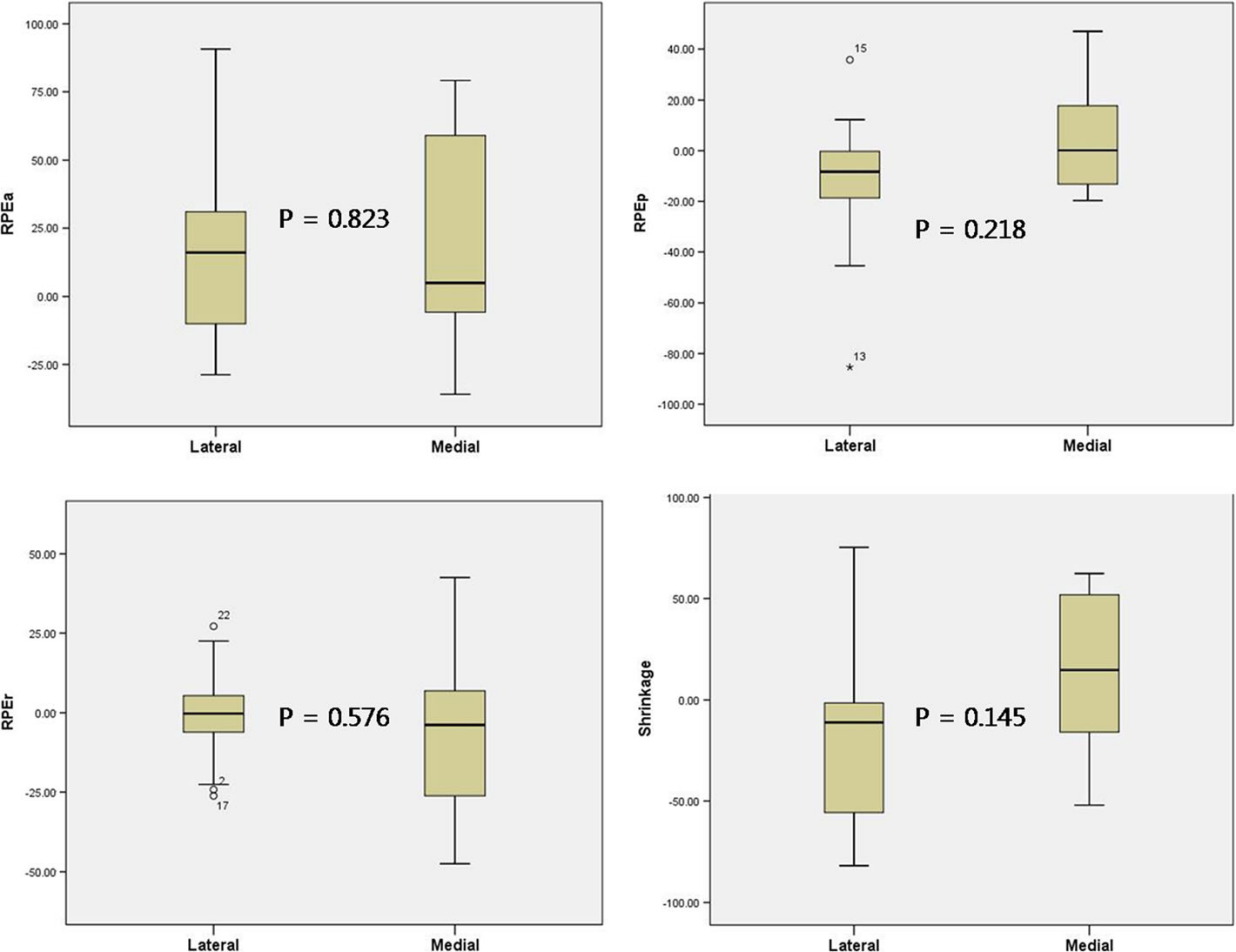
Comparison of extrusion and shrinkage progression between medial and lateral meniscal allograft transplantation

The difference in RPE changes and shrinkage between 6 medial and 24 lateral MATs were compared. There was no statistical difference in progression of anterior (*P* = 0.823), posterior (*P* = 0.218), and radial (*P* = 0.576) extrusion between the medial and lateral directions. Shrinkage was also statistically insignificant (*P* = 0.145) (Fig. 2).

RPE in radial (*R* = 0.147, *P* = 0.525), anterior (*R* = 0.249, *P* = 0.264), and posterior (*R* = 0.230, *P* = 0.315) directions and shrinkage (*R* = 0.176, *P* = 0.435) were not correlated to IKDC scores at postoperative 12 months. Possible confounding factors that can influence the clinical outcome including age, sex, body mass index, laterality of MAT (medial or lateral), or time from previous meniscectomy did not show any significant correlation.

## Discussion

In this study, the graft extrusion and shrinkage did not progress between 3 months and 1 year period after MAT in both coronal and sagittal planes. In addition, shrinkage and extrusion of the graft were not related to early clinical outcomes of MAT.

Graft shrinkage is a potential complication of MAT and understood as a change in the graft property during the remodeling process, and several studies have shown that meniscal allografts lose normal collagen architecture during early remodeling periods, and a loss of microarchitecture may cause morphologic alterations [13, 21, 22]. Although it has been regarded as a less frequent problem with the advent of the current (deep-frozen) preservation technique, few studies reported the shrinkage still occurs with deep-frozen grafts [21]. Moreover, studies on the effect of graft shrinkage on the clinical outcome after MAT are surprisingly rare, and it is still unclear whether the graft shrinkage is a progressive phenomenon. A laboratory study by Dienst et al. [14] showed that undersized graft causes increased forces across the articular cartilage and allograft itself. A serial MRI study by Lee et al. [23] reported that although 65% of cases showed graft shrinkage, it occurred during the first 3 months but stabilized thereafter. Another serial MRI study by Carter and Economopoulos [24] reported that graft shrinkage was observed until 6 months after MAT. In our study, there was no significant progression of shrinkage after 3 months and shrinkage showed no effect on the clinical outcome after 1 year of surgery. This finding suggests that shrinkage may be an initial or early phenomenon after surgery caused by micro-architectural remodeling, but does not progress after the remodeling ends.

The extrusion of the graft is regarded as another complication after MAT, and relatively more studies have investigated this issue compared to the shrinkage. Several studies have investigated the change of graft extrusion after MAT using serial MRI. Lee et al. [25] found that the graft extrusion occurred in early (< 6 weeks) after MAT, while it did not progress until postoperative 1 year. Recently, a mid-term follow-up study reported that early (< 6 weeks) graft extrusion did not progress until 3- to 5-year MRI follow-up [26]. In addition, Ha et al. [27] reported no significant difference in graft extrusion between 1 and 4 years after MAT. It is noteworthy that the graft extrusion was evaluated from both coronal and sagittal MRI images. Most of the studies focused on the graft extrusion in radial direction observed on coronal plane, while only few studies from the same study group [26, 28] evaluated the graft extrusion in anterior or posterior direction. In our study, the graft extrusion in both coronal and sagittal directions did not progress between 3 months and 1 year post-MAT. Based on this finding, it is supposed that the graft “extrusion” may be not a progressive problem, but rather may be a problem that already existed immediately after surgery due to assumed reasons including graft size mismatch or inadequate graft position. There were studies on efforts to prevent graft extrusion by reducing the graft size [29] or using different approach for graft placement [30–32]. In this respect, the authors recently modified medial MAT technique as tensioning the anterior horn of the graft using suture anchor after bone fixation (unpublished data).

Association between the extrusion of native meniscus and progression of symptomatic knee osteoarthritis has been reported [33–35]. However, many studies on the extrusion after MAT reported that the extent of extrusion did not correlate with the clinical outcomes, although they have confirmed the occurrence of extrusion [26, 28, 36, 37]. Our study result is in accordance with previous MAT studies that even though the graft extrusion was observed, there was no significant correlation between the extrusion and the clinical outcome.

The difference in progression of extrusion and shrinkage between the medial and lateral MAT cases was also compared, and no significant difference was found out. Although there were reports showing more graft extrusion after medial MAT compared to lateral MAT [28, 38], it has not been studied if there are any differences in the progression of extrusion or shrinkage between lateral and medial MATs. However, this issue was not one of our study purposes and our finding cannot be generalized since it may be underpowered with considerably different number of lateral and medial MAT cases.

This study has some limitations. First, the follow-up period was relatively short to evaluate the long-term clinical outcomes of the procedure. Second, the possible early change of the graft within weeks after MAT could not be evaluated, since MRI was first taken 3 months after the surgery. It would be beneficial as reference if immediate postoperative MRI was available. However, it was concerned that early postoperative changes including increased joint effusion, soft tissue swelling may obscure accurate MRI evaluation. In addition, it may be difficult for patient to maintain an adequate position to take MRI at immediate postoperative period. Third, the accuracy of determining the meniscal margin on sagittal images is known to be lower than the measurement from coronal images [39]. Four observers measured all parameters in same, predetermined sections to compensate this possible error, and inter- and intra-observer reliabilities were excellent in our study.

## Conclusion

In the coronal and sagittal planes, extrusion and shrinkage did not progress from 3 months to 1 year after meniscal allograft transplantation. This finding suggests that graft extrusion or shrinkage is not progressive, but rather is a static phenomenon. Not the characteristics of the graft itself, but the techniques for graft sizing, fixation, and positioning may be determinants of the early graft change after MAT. In addition, shrinkage and extrusion of the graft did not correlate with early clinical outcomes of MAT. Therefore, multiple, serial MRI may be not necessary in early period after MAT, even though the graft extrusion or shrinkage occurs. However, future studies will be required to determine the effects of the graft extrusion or shrinkage on long-term clinical outcome of MAT.

## Abbreviations

ICC: Intra-class correlation coefficient
IKDC: International Knee Documentation Committee
MAT: Meniscus allograft transplantation
MRI: Magnetic resonance imaging
RPE: Relative percentage of extrusion
RPEa: Relative percentage of extrusion of anterior direction
RPEp: Relative percentage of extrusion of posterior direction
RPEr: Relative percentage of extrusion of radial direction
